# Effect of bovine milk fermented with *Lactobacillus rhamnosus* L8020 on periodontal disease in individuals with intellectual disability: a randomized clinical trial

**DOI:** 10.1590/1678-7757-2018-0564

**Published:** 2019-07-29

**Authors:** ODA Yuki, Chiaki FURUTANI, Yuika MIZOTA, Atsuko WAKITA, Sumiyo MIMURA, Takuya KIHARA, Masaru OHARA, Yoshiyuki OKADA, Mitsugi OKADA, Hiroki NIKAWA

**Affiliations:** 1 Hiroshima University Hospital, Department of Special Care Dentistry, Hiroshima, Japan.; 2 Hiroshima University Hospital, Department of Clinical Practice and Support, Division of Dental Hygiene, Hiroshima, Japan.; 3 Hiroshima University, Department of Oral Biology and Engineering, Hiroshima, Japan.; 4 Tsurumi University, School of Dental Medicine, Department of Fixed Prosthodontics, Yokohama, Japan.; 5 Hiroshima University Hospital, Department of Advanced General Dentistry, Hiroshima, Japan.

**Keywords:** Milk, Intellectual disability, Lactobacillus, Probiotics, Yogurt, Periodontal diseases

## Abstract

Studies on the oral health of individuals with intellectual disability (ID) have identified problems that include a high prevalence of periodontal disease. The use of probiotics to treat periodontal disease has been the focus of considerable research, and bovine milk fermented with *Lactobacillus rhamnosus* L8020 (L8020 yogurt) has been shown to reduce the oral prevalence of four periodontal pathogens.

**Objective:**

The aim of this randomized, double-blind, placebo-controlled trial was to compare the effects of L8020 yogurt (test group) with those of placebo yogurt (placebo group) on the papillary-marginal-attached (PMA) index, gingival index (GI), and probing depth (PD) in 23 individuals with ID.

**Methodology:**

All patients were required to consume the allocated yogurt after breakfast for 90 days. PMA index and GI scores as well as PDs were assessed before the start of yogurt consumption (baseline), after 45 and 90 days of consumption, and 30 days after the cessation of consumption. Student’s t-test, Mann–Whitney U test or Fisher’s exact test was used for inter-group comparisons, and the mixed effect model of repeated measurements was used for data analysis.

**Results:**

The decrease in PMA index score was significantly greater in the test group than in the placebo group (p<0.001). The GI score also decreased during the study, with a tendency for greater decrease in the test group. Furthermore, decreases in PD between baseline, 45 and 90 days tended to be greater in the test group than in the placebo group.

**Conclusion:**

These results suggest that regular consumption of bovine milk fermented with *L. rhamnosus* L8020 can lower the risk of periodontal disease in individuals with ID.

## Introduction

Intellectual disability (ID) is defined as sub-average general intellectual function and is associated with impaired adaptive behavior.^1^ Many researchers have reported that individuals with ID have worse oral hygiene and higher rates of dental disease than ordinary individuals.^2-4^ Reported oral health-related problems in individuals with ID include poor oral hygiene, progressed dental caries, and a high prevalence of periodontal disease.^5^ The oral health status of these patients is affected by their patterns of cognition, developmental age,^6^ and other factors such as age, the personality and compliance of the caregiver, and the presence of underlying diseases, including physical disabilities.^2^ Individuals with ID experience difficulty in understanding the importance of oral health and tend to co-operate less for oral cleaning procedures. Because maintenance of a healthy oral environment in patients with ID is not easy,^7^ available measures for the prevention of oral disease and improvement of oral hygiene in these individuals are limited,^8^ although necessary.^9^

The Food and Agriculture Organization/World Health Organization defines probiotics as live microorganisms that confer a health benefit to the host (i.e. improve the microbial balance in the intestinal tract) when consumed in adequate amounts (in food or as dietary supplements).^10,11^ The use of probiotics for the treatment of periodontal disease has been the focus of considerable research.^12,13^ Organisms commonly associated with periodontal disease include *Porphyromonas gingivalis*, *Prevotella intermedia*, *Tannerella forsythia*, *Dialister pneumosintes*, *Campylobacter rectus*, *Fusobacterium* spp, *Selenomonas sputigena*, *Parvimonas micra*, and spirochetes such as *Treponema denticola*.^14^ Nikawa, et al.^15^ (2004) demonstrated that yogurt containing *Lactobacillus rhamnosus* L8020 (L8020 yogurt), which has been detected as a normal strain of bacteria in the oral cavity,^16^ significantly lowered the risk of dental caries, suggesting that daily consumption of this probiotic organism may be useful in the prevention of caries. *L. rhamnosus* L8020 is also known to be active against a broad spectrum of bacteria that cause periodontal disease.^16^ Specifically, L8020 yogurt has been shown to decrease the oral carriage of four known periodontal pathogens, namely *P. gingivalis*, *P. intermedia*, *T. forsythia*, and *Fusobacterium* spp.^16^

Although mechanical removal of plaque biofilm by scaling and root planing is an essential aspect of periodontal therapy, the complete removal of plaque-retentive factors is not always possible,^17^ particularly for individuals with ID, who are mostly noncompliant with dental treatment. The use of probiotics to promote oral health has been reported in a few studies,^18,19^ with the probiotics used being mostly derived from the intestine. However, data on the effects of probiotic bacteria on periodontal pathogens are limited.^16^ The aim of this randomized controlled trial was to determine the effect of L8020 yogurt on the papillary-marginal-attached (PMA) index,^20^ gingival index (GI),^21^ and probing depth (PD) in individuals with ID. The PMA index evaluates gingival inflammation according to the degree of involvement of the interdental papilla as well as the marginal and attached gingiva, where GI is the number assigned to designate the degree of gingival inflammation. PD precisely assesses the severity of periodontal disease through measurement of the periodontal pocket depth using a periodontal probe.

## Methodology

### Study design

**Figure 1:**
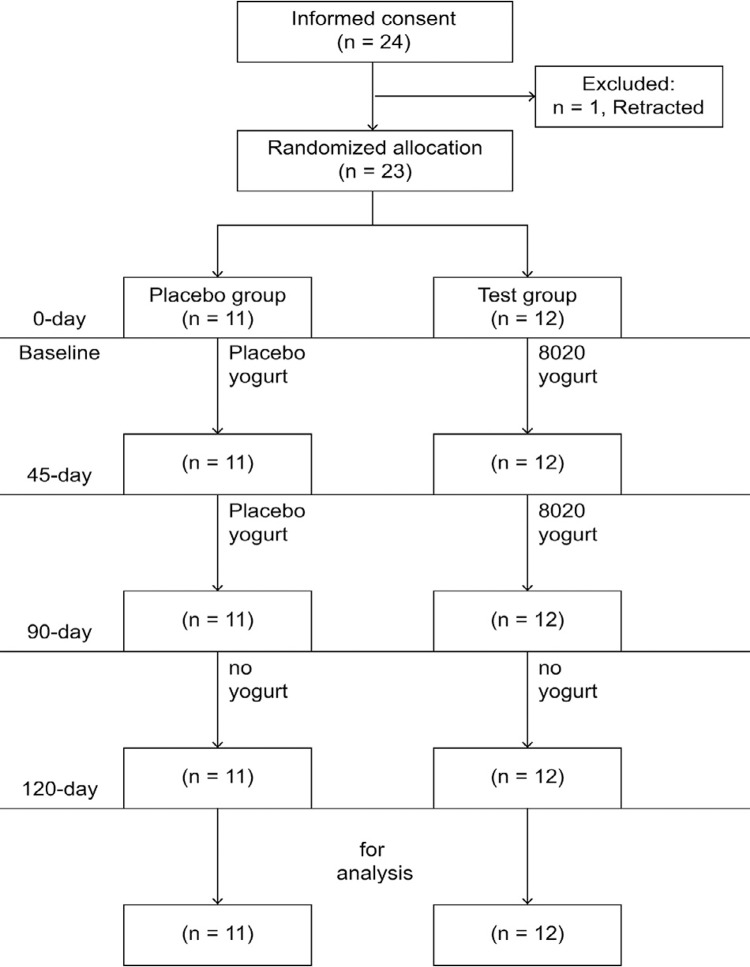
Study flowchart. Individuals with intellectual disability were allocated to receive L8020 yogurt (bovine milk fermented with *Lactobacillus rhamnosus* L8020; test group) or a conventionally prepared commercial yogurt (placebo group). The study parameters were measured before the start of yogurt consumption (baseline), after 45 and 90 days of continuous daily yogurt consumption, and 30 days after cessation of yogurt consumption

Twenty-three outpatients with ID were enrolled in this randomized, double-blind, placebo-controlled trial. The patients were randomly allocated to a L8020 yogurt group (n=12) or a placebo yogurt group (n=11, after one patient withdrew consent before randomization; Figure 1). All patients were required to consume 80 g of the allocated yogurt after breakfast in the morning for 90 days. During this time, they were instructed to refrain from toothbrushing or consuming any food or beverage for 1 h after consumption of the yogurt. The patients were assessed at baseline (before the start of daily yogurt consumption), after 45 and 90 days of yogurt consumption, and 30 days after the cessation of yogurt consumption (120 days after the start of consumption). Simple randomization and centralized allocation methods were used. A random number table was generated by an off-site computer (Shikoku Nyugyou, Co. Inc, Toon City, Ehime Prefecture, Japan), and the assignment table was prepared by the research manager (HN) using the random number table. Shikoku Nyugyou, Corp. was responsible for the assignment. The intervention materials (L8020 yogurt and placebo yogurt) were created, surveyed, individually packed in a quantity of 80 g (as is the commonly used amount for yogurt in Japan) and delivered under the control of Shikoku Nyugyou, Corp. The dentists and research staff at the trial site were blinded to the yogurt preparation used during the study procedures.

### Study participants

The 23 study participants (17 men, six women) had severe ID (intelligence quotient, 35–50). Their mean age was 33.2 (range, 20–45) years, and the mean periodontal pocket depth was <6 mm. All patients had permanent dentition and visited a Hospital in the city of Hiroshima. For inclusion criteria, patients had finished active treatment for dental disease and visited the clinic for regular check-ups involving oral examinations. All patients in this study had regular check-ups for 10.00±5.31 (range, 1–19) years and had received periodontal examination. As for the exclusion criteria, their hospital records for 2015–2017 were reviewed to confirm the absence of conditions that can influence the occurrence of periodontal disease, such as diabetes, autoimmune diseases and infectious diseases like AIDS. Patients who showed extremely uncooperative attitudes toward periodontal examinations were also excluded. Individuals who had received antibiotics for the last 3 months and those who already consumed yogurt on a daily basis were also excluded. None of the patients had active caries, smoked, or were on medication. The study protocol was approved by the Ethical Committee of the University (Epidemiology-No. E-342), and the study was registered in the University Hospital Medical Information Network Center (UMIN) under ID 000023503. Consent for participation was obtained from at least one guardian for each patient after explanation of the purpose and contents of this study, in accordance with the ethical guidelines of the Declaration of Helsinki (1975). At the same time, participants were told that they could withdraw their consent at given moment for any reason.

### Bacterial strains in the trial yogurt preparations

L8020 yogurt was prepared by the addition of *Streptococcus thermophilus* to a large amount of frozen stock culture of *L. rhamnosus* L8020. *L. bulgaricus* and *S. thermophilus*, which are widely used in fermented milk products in Japan, were used for preparation of the placebo yogurt. Standard methods were used for fermentation of the test and placebo samples.^16^ The L8020 yogurt contained approximately 1.5×10^9^lactic acid bacteria, with 2.0×10^7^ L8020 cells per gram. The placebo yogurt contained approximately 1.5×10^9^ lactic acid bacteria. The final pH values for the L8020 yogurt and placebo yogurt were 4.35 and 4.37, respectively.

### Measurements

Dental examinations and periodontal examinations were performed by a single dentist in the Special Care Dental Clinic, under artificial lighting with the patient supine in a dental chair. The PMA index is assigned to each gingival unit of the maxillary and mandibular anterior teeth as follows: 0, no inflammation and 1, inflammation. The total PMA index score was calculated for each patient accordingly. This index evaluates the number of gingival units (papillary, marginal, and attached gingiva) exhibiting gingival inflammation around erupted teeth and is limited to the anterior gingiva. It is assessed by observation without the use of a prescribed instrument. We also calculated the GI score for all patients on the basis of the following scoring system: 0, no inflammation; 1, mild inflammation; 2, moderate inflammation; and 3, severe inflammation. This was assessed using a mouth mirror and a calibrated periodontal probe (UNC-15, Hu-Friedy, Mfg. Co., LLC, Chicago, IL, USA). PD was measured from the gingival margin to the bottom of the periodontal pocket using a mouth mirror and a calibrated periodontal probe (UNC-15, Hu-Friedy, Mfg. Co., LLC, Chicago, IL, USA). For each patient, GI and PD were measured at the mesiobuccal, distobuccal, mesiolingual and distolingual aspects of the maxillary right first molar, maxillary right lateral incisor, maxillary left first premolar, mandibular right first premolar, mandibular left lateral incisor, and mandibular left first molar. The mean GI score and PD were calculated from the values for these 24 points.

### Intraexaminer reliability

Ten patients who were not participants in this study, each of whom had two contralateral teeth with a PD of >6 mm, were used for assessment of reproducibility. The examiner evaluated these patients on three occasions separated by a 3-day interval. The reproducibility of PD measurements was sufficiently high (grand mean: 2.2, change in mean: 0.07, typical error: 0.30, repeatability coefficient: 0.84, and intraclass correlation coefficient: 0.63). This evaluation procedure was periodically repeated during the study.

### Statistical analysis

The data were analyzed using the full analysis set. Continuous variables that were normally distributed are expressed as means and standard errors, while those with non-normal distribution are expressed as medians and interquartile ranges. Categorical variables are expressed as numbers and percentages. The baseline characteristics of the two groups were compared using Student’s *t*-test (for continuous variables with normal distribution), Mann–Whitney *U* test (for variables with non-normal distribution), or Fisher’s exact test (for nominal variables). The mixed effect model repeated measurement (MMRM)^22,23^ has been extensively used for the analysis of data for continuous endpoints in longitudinal clinical trials. We also used MMRM, with patients as a random factor (compound symmetry correlation structure) and time and group (including the interaction factor) as a fixed factor in order to determine the longitudinal effect of continuous consumption of L8020 yogurt on the PMA index, GI, and PD. All statistical analyses were performed using JMP Pro 12 (SAS Institute Japan Inc, Tokyo, Japan). A *P*-value of <0.05 was considered statistically significant.

## Results

Written informed consent was obtained for 24 patients before randomization. Consent was withdrawn for one patient who was not accustomed to eating breakfast every day. Twenty-three patients completed the study after randomization to the test (n=12) and placebo (n=11) groups and were consequently included in our analysis (Figure 1). There were no significant differences in the baseline characteristics of patients between the two groups (Table 1).

**Table 1:** Baseline demographic and dental characteristics of individuals with intellectual disability

		Placebo group			Test group			p-value
n		11			12			-
Sex, n (%)								0.999†
	Male	8		-72.7	9		-75	
	Female	3		-27.3	3		-25	
Age, years (mean ± SD)		32.9	±	6.3	33.4	±	4.5	0.825‡
Disability, n (%)								
	Mental retardation	11		-100	12		-100	>0.999†
	Autistic spectrum disorder	4		-36.4	6		-50	0.680†
	Down syndrome	0		0	0		0	>0.999†
	Cerebral palsy	1		-9.1	0		0	0.478†
	Epilepsy	1		-9.1	3		-25	0.590†
Number of teeth, median (IQR)		28	(26, 28)		28	(25, 28)		0.880§
DMFT		9.2	±	7.9	9.6	±	6.5	0.895‡
Baseline PMA index		21.5	±	8.6	24.8	±	6.9	0.336‡
Baseline GI		1.59	±	0.31	1.55	±	0.24	0.761‡
Baseline PD		2.61	±	0.39	2.9	±	0.66	0.220‡

Placebo group: Patients consumed placebo yogurt for 90 days

Test group: Patients consumed bovine milk fermented with *Lactobacillus rhamnosus* L8020 (L8020 yogurt) for 90 days

†Fisher’s exact test, ‡unpaired t-test, §Mann–Whitney U test

Values are expressed as means and standard deviations unless otherwise stated.

DMFT: decayed, missing, and filled teeth; GI: gingival index; SD: standard deviation; IQR: interquartile range; PD: probing depth; PMA: papillary-marginal-attached

**Figure 2:**
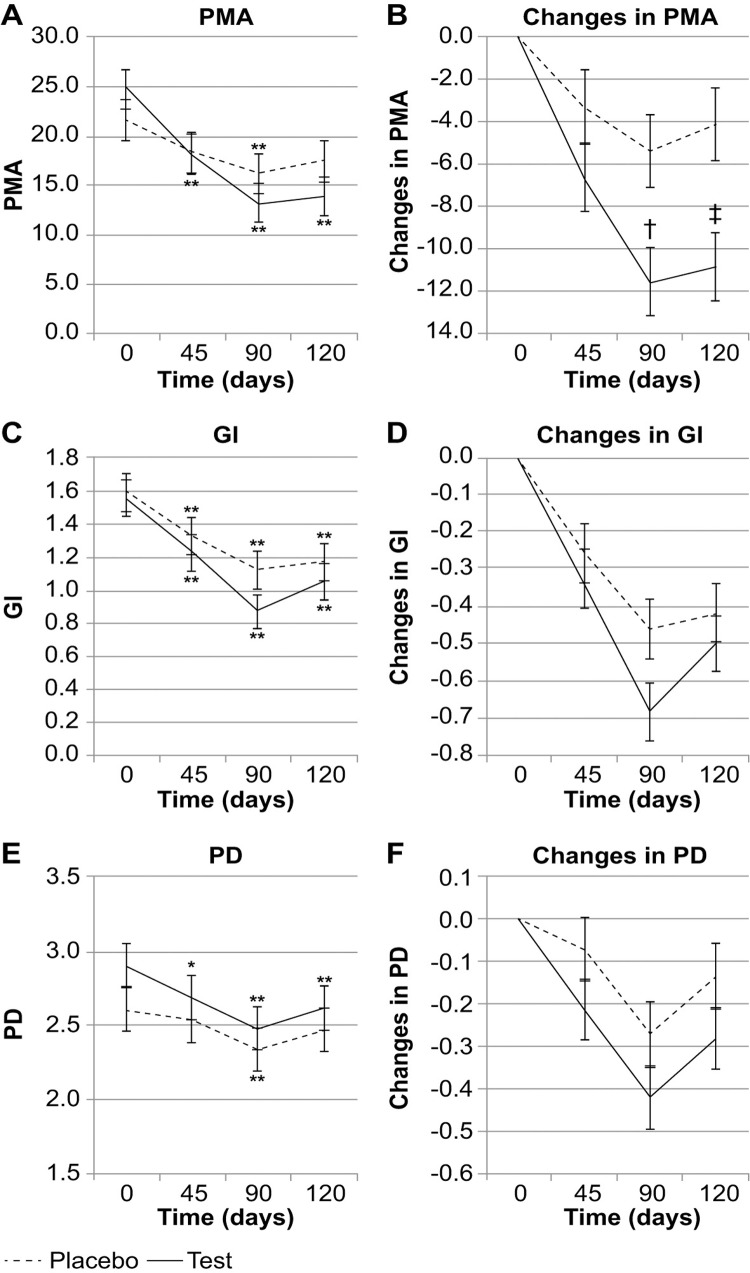
Mean values and magnitude of change in each clinical parameter for individuals with intellectual disability who consumed L8020 yogurt (bovine milk fermented with *Lactobacillus rhamnosus* L8020; test group) or a conventionally prepared commercial yogurt (placebo group). (A) Mean PMA index scores at baseline, after 45 and 90 days of continuous daily yogurt consumption, and 30 days after the cessation of yogurt consumption (120 days after the start of consumption); (B) The magnitude of change in the PMA index score from baseline to 90 days and from baseline to 120 days is significantly greater in the test group than in the placebo group (p = 0.006); (C) Mean GI scores at baseline, after 45 and 90 days of continuous daily yogurt consumption, and 30 days after cessation of yogurt consumption; (D) Magnitude of change in the GI score from baseline to 90 days and from baseline to 120 days; (E) Mean PD values at baseline, after 45 and 90 days of continuous daily yogurt consumption, and 30 days after cessation of yogurt consumption; (F) The magnitude of change in PD from baseline to 90 days and from baseline to 120 days. GI, gingival index; PD, probing depth; PMA, papillary-marginal-attached

The mean (standard error) PMA index score in the test group decreased significantly, from 24.8 (2.0) at baseline to 18.1 (2.0) after 45 days of yogurt consumption, 13.2 (2.0) after 90 days of consumption, and 13.9 (2.0) at 30 days after the cessation of consumption (*p*<0.001 vs. baseline for all). The corresponding values in the placebo group were 21.5 (2.1), 18.3 (2.1) (*p*=0.177), 16.2 (2.1) (*p*=0.007), and 17.5 (2.1) (*p*=0.057), respectively. Thus, there was no significant difference between the baseline value and the value at 30 days after cessation of consumption (Figure 2A). After yogurt consumption for 90 days, there was a significant difference in the PMA index score between the two groups (time*group factor: *p*=0.022). The mean decreases in the PMA index score from baseline to 90 days and from baseline to 120 days were significantly greater in the test group than in the placebo group [−11.6 (1.6) vs. −5.4 (1.7), *p*=0.010; −10.8 (1.6) vs. −4.1 (1.6), *p*=0.006, respectively; Figure 2B].

Data are expressed as means ± standard errors of the mean

*p<0.05, **p<0.01 vs. time 0; †p<0.05, ‡p<0.01 vs. placebo

The mean GI score for the test group showed a significant decrease from 1.55 (0.11) at baseline to 1.22 (0.11) after 45 days of consumption, 0.87 (0.11) after 90 days of consumption, and 1.05 (0.11) at 120 days after the start of consumption (*p*<0.001 vs. baseline for all). The placebo group also showed a significant decrease in the mean GI score, from 1.59 (0.12) at baseline to 1.33 (0.12) at 45 days (*p*=0.006), 1.13 (0.12) at 90 days (*p*<0.001), and 1.17 (0.12) at 120 days (*p*<0.001; Figure 2C). After yogurt consumption for 90 days, there was no significant difference in the GI score between the two groups (time*group factor, *p*=0.255). However, the mean decrease in the score from baseline to 90 days tended to be greater in the test group than in the placebo group [−0.68 (0.08) vs. −0.46 (0.08); *p*=0.050; Figure 2D].

The mean PD in the test group showed a significant decrease from 2.90 (0.14) mm at baseline to 2.68 (0.14) mm at 45 days (*p*=0.015), 2.48 (0.14) mm at 90 days (*p*<0.001), and 2.62 (0.14) mm at 120 days (*p*<0.001). In the placebo group, the mean PDs at baseline and 45 and 90 days were 2.61 (0.15), 2.53 (0.15) (*p*=0.999), and 2.33 (0.15) mm (*p*=0.002), respectively. Thereafter, mean PD increased to 2.47 (0.15) mm at 120 days (*p*=0.234), and there was no significant difference from the baseline depth at this time point (Figure 2E). After yogurt consumption for 90 days, there was no significant difference in PD values between both groups (time*group factor: *p*=0.428), although the mean decrease at 45, 90, and 120 days tended to be greater in the test group than in the placebo group [−0.21 (0.07) vs. −0.07 (0.08), *p*=0.189; −0.42 (0.07) vs. −0.27 (0.08), *p*=0.167; and −0.28 (0.07) vs. −0.14 (0.08), *p*=0.174, respectively; Figure 2F].

## Discussion

In this study, we found that regular consumption of bovine milk fermented with *L. rhamnosus* L8020 (L8020 yogurt) can lower the risk of periodontal disease in individuals with ID.

The number of individuals with ID is increasing worldwide, including Japan, because of population growth, increased reporting and more accurate methods for detection and diagnosis.^24^ The Summary of the Annual Report on Governmental Measures for Persons with Disabilities published by the Cabinet Office of Japan in 2016 reported that the population with ID in Japan doubled from 297,100 in 1995 to 622,000 in 2011.^25^ Many ID-related characteristics may increase the risk of periodontal disease;^26^ therefore, individuals with ID may have worse oral hygiene and more oral disease than do healthy individuals.^2-4^ The ID population is very heterogeneous, ranging from individuals with mild ID, who should be fully integrated into the general population in every aspect, to those with severe ID, who need full-time care. Therefore, the specific oral health care needs of the subgroups in this population require clear definition.^5^ Ozgul, et al.^27^ (2014) reported a significant positive correlation of the severity of ID with clinical periodontal indices and the number of missing teeth. Clearly, toothbrushing is the most effective way of preventing periodontal disease. However, our study sample was limited to patients with severe ID, for whom toothbrushing and oral cleaning by caregivers may be impossible due to noncompliance. Thus, other measures for preventing periodontal disease that are easily accepted by individuals with severe ID would be beneficial.

Although our patient sample comprised individuals with ID, it should be noted that even elderly individuals who need full-time care cannot brush their teeth on their own. Therefore, L8020 yogurt consumption may be a useful strategy for the maintenance of oral health in elderly individuals as well. Future studies should explore the effect of L8020 yogurt consumption on periodontal diseases in the elderly.

In Japan, there are records of 31.90 million individuals aged ≥65 years. The proportion of individuals in this age group increased from 24.1% in 2012 to 25.1% in 2013, and a certain projection states that one in 2.5 individuals and one in four individuals will be aged ≥65 years and ≥75 years by 2060.^28^ In the future, as the overall population of Japan decreases, the proportion of elderly individuals requiring assistance with activities of daily living, including toothbrushing, will continue to increase, so an effective strategy for the prevention of periodontal disease in this population would be welcomed.

Evidence supporting the health benefits of probiotics is steadily accumulating, and new therapeutic horizons, particularly those related to *Lactobacillus* spp. and Bifidobacteria, are emerging in the field of periodontal disease.^29^ Recent studies have demonstrated the effectiveness of probiotics containing *Lactobacillus* spp. in the prevention of periodontal disease;^29,30^ however, there is limited research on the clinical benefits of *L. rhamnosus* L8020 (L8020 yogurt). Although Nikawa, et al.^16^ (2011) reported that L8020 yogurt reduced the carriage of *P. gingivalis*, *P. intermedia*, *T. forsythia*, and *Fusobacterium* spp. *in vitro*, it remained unclear whether clinical benefits would be seen in patients as well. In our study, the magnitude of change in the mean PMA score from baseline to 90 and 120 days after the start of daily L8020 yogurt consumption was significantly greater in the test group than in the placebo group. Furthermore, the magnitude of change in the GI score and PD from baseline to 90 days tended to be greater in the test group than in the placebo group, suggesting that L8020 yogurt has a beneficial effect on periodontal disease in patients with ID. Although changes in all three parameters showed a decreasing trend, only the change in the PMA index score decreased significantly after 90 days of consumption of L8020 yogurt. PMA differs from the other two parameters in that it reflects inflammation of the papillary, marginal, and attached gingiva, rather than reflecting only inflammation of the periodontal pockets. Thus, it is possible that a decrease in periodontal pathogens affects inflammation of the papillary, marginal, and attached gingiva earlier than that of the periodontal pockets. However, further investigation is needed to determine the order in which probiotics exert their clinical effects on the periodontal gingiva.

In our study, there was a decrease in PD after 90 days of continuous yogurt consumption in both experimental groups. We speculate that there may be two explanations for the decrease in PD in the patients who consumed the placebo yogurt that did not contain *L. rhamnosus* L8020. First, all study participants were instructed to refrain from toothbrushing or consuming any food or beverage for 1 h after consumption of the allocated yogurt in order to maximize the amount of time for which the probiotic bacteria in the L8020 yogurt remained in the oral cavity. Also, the participants’ guardians or caregivers were instructed to brush their teeth at a specific time in the morning before yogurt consumption, so daily toothbrushing was performed for all participants in both study groups, including those who were not accustomed to it. Second, *Lactobacillus* strains are commonly used probiotics,^16^ and it is possible that *Lactobacillus* spp. contained in the placebo yogurt also influenced periodontal bacteria. Further research using next-generation sequencing is necessary to elucidate the changes that occur in the oral microbiota in response to continuous consumption of yogurt containing different types of probiotics.

This study has some potential limitations, including the limited observation period of 120 days, the relatively short yogurt consumption period of 90 days, and the small sample size. As this study was a preliminary rather than a confirmation study investigating the clinical effect of L8020 yogurt, there was no previous information with which to determine an adequate sample size. On the basis of a similar previous study,^29^we calculated that 15 individuals with ID who were otherwise healthy would be required to ensure an adequate sample size. However, our trial was performed on patients with severe ID, and we were thus limited in the recruitment of participants who met the study criteria. In particular, among the 1109 outpatients who regularly visit our office in the Hiroshima area, it was extremely difficult to find individuals with severe ID who were available for irregular visits and repeated dental examinations. Hence, we decided that the final sample size was within the realistic and feasible range. Though we were unable to fulfill the initial goal of 15 subjects, in retrospect, the amount of change in PMA after the start of intervention was -10.8±5.6 (mean±SD) in the test group and -4.1±5.3 in the placebo group, meaning that our sample size was appropriate for determining differences between the two groups with a power of 80.2%. The preliminary results presented here remain to be confirmed in additional subjects in a future study.

Although probiotics in the microbiota of the human oral cavity, if compatible with the ecosystem, are likely to be effective in preventing periodontal disease,^29^ the minimum duration of consumption before the development of changes in the oral microbiota remains unclear. The difference in PD between baseline and 45 days and between baseline and 90 days in our test group indicates that PD decreases with an increase in the duration of yogurt consumption; however, it remains unknown whether long-term consumption of L8020 yogurt has additional benefits or a ceiling effect. Therefore, toothbrushing remains the most important preventive and therapeutic strategy for periodontal disease, and probiotics should only be used as a supplementary measure. Patients, parents, and caregivers should be warned that the consumption of L8020 yogurt alone cannot replace toothbrushing as a measure for the maintenance of oral health.

## Conclusions

In conclusion, the consumption of bovine milk-derived *L. rhamnosus* L8020 yogurt for 90 days resulted in significantly greater changes in the PMA index than the consumption of placebo yogurt for the same duration; moreover, it tended to decrease GI score and PD. Our findings suggest that *L. rhamnosus* L8020 may be a useful probiotic organism when included in products that are ingested daily. However, further investigation is needed to elucidate the effect of *L. rhamnosus* L8020 on the oral microbiota and clarify the limits of the effects of this probiotic organism on periodontal disease.

### Ethical approval

The study protocol was approved by the Ethical Committee of Hiroshima University (Epidemiology-No. E-342), and the study has been registered in the University Hospital Medical Information Network (UMIN) Center (ID 000023503). Consent for participation was obtained from at least one guardian of each patient in accordance with the ethical guidelines of the Declaration of Helsinki (1975).
